# Gemcitabine Plus Erlotinib for Advanced Pancreatic Cancer: A Systematic Review with Meta-Analysis

**DOI:** 10.1371/journal.pone.0057528

**Published:** 2013-03-05

**Authors:** Zu-Yao Yang, Jin-Qiu Yuan, Meng-Yang Di, Da-Yong Zheng, Jin-Zhang Chen, Hong Ding, Xin-Yin Wu, Ya-Fang Huang, Chen Mao, Jin-Ling Tang

**Affiliations:** 1 Division of Epidemiology, The Jockey Club School of Public Health and Primary Care, The Chinese University of Hong Kong, Hong Kong, China; 2 The Hong Kong Branch of The Chinese Cochrane Centre, The Chinese University of Hong Kong, Hong Kong, China; 3 Department of Oncology, Nanfang Hospital, Southern Medical University, Guangzhou, Guangdong Province, China; 4 Department of Epidemiology, School of Public Health and Tropical Medicine, Southern Medical University, Guangzhou, Guangdong Province, China; 5 Shenzhen Municipal Key Laboratory for Health Risk Analysis, Shenzhen Research Institute of The Chinese University of Hong Kong, Shenzhen, Guangdong Province, China; Sapienza University of Rome, Italy

## Abstract

**Background:**

This study aims to comprehensively summarize the currently available evidences on the efficacy and safety of gemcitabine plus erlotinib for treating advanced pancreatic cancer.

**Methodology/Principal Findings:**

PubMed, EMBASE, The Cochrane Library and abstracts of recent major conferences were systematically searched to identify relevant publications. Studies that were conducted in advanced pancreatic cancer patients treated with gemcitabine plus erlotinib (with or without comparison with gemcitabine alone) and reporting objective response rate, disease control rate, progression-free survival, time-to-progression, overall survival, 1-year survival rate and/or adverse events were included. Data on objective response rate, disease control rate, 1-year survival rate and adverse events rate, respectively, were combined mainly by using Meta-Analyst software with a random-effects model. Data on progression-free survival, time-to-progression and overall survival were summarized descriptively. Sixteen studies containing 1,308 advanced pancreatic cancer patients treated with gemcitabine plus erlotinib were included. The reported median progression-free survival (or time-to-progression), median overall survival, 1-year survival rates, objective response rates and disease control rates were 2–9.6 months, 5–12.5 months, 20%–51%, 0%–28.6% and 25.0%–83.3%, respectively. The weighted 1-year survival rate, objective response rate and disease control rate based on studies reporting robust results were 27.9%, 9.1% and 57.0%, respectively. According to the studies with relevant data, the incidences of total and severe adverse events were 96.3% and 62.9%, respectively. The most frequently reported adverse events were leucopenia, rash, diarrhea, vomitting, neutropenia, thrombocytopenia, anaemia, stomatitis, drug-induced liver injury, fatigue and fever. Compared with gemcitabine alone, the progression-free survival and overall survival with gemcitabine plus erlotinib were significantly longer, but there were also more deaths and interstitial lung disease-like syndrome related to this treatment.

**Conclusions/Significance:**

Gemcitabine plus erlotinib represent a new option for the treatment of advanced pancreatic cancer, with mild but clinically meaningful additive efficacy compared with gemcitabine alone. Its safety profile is generally acceptable, although careful management is needed for some specific adverse events.

## Introduction

As a highly malignant disease, pancreatic cancer is the eighth, fourth and fifth leading cause of cancer-related deaths in the world, the United States and Europe, respectively [1]–[5]. More than 80% of the new cases have either locally advanced or metastatic disease, which is often referred to as advanced pancreatic cancer (APC), at the time of diagnosis [1], [6]. Without treatment, the length of survival with APC is only about 2 to 4 months [7]. Gemcitabine (GEM), a nucleoside analog under the trade name “Gemzar”, is effective in the treatment of APC in terms of both response rate and median overall survival [8]. However, the benefit it brings is modest, and it does not improve the dismal prognosis much, with a median overall survival of less than 6 months [8].

Various cytotoxic chemotherapy drugs such as 5-fluorouracil, cisplatin, oxaliplatin, irinotecan, pemetrexed and capecitabine, in combination with GEM, have been investigated as alternative options for the treatment of APC. However, they failed to improve the overall survival of patients significantly, although the progression-free survival, time-to-progression and/or objective response rate could be increased to varying degrees [9]–[14]. Hence, there is a continuous need for more effective drugs that can be used alone or together with existing chemotherapies to further improve the prognosis of APC.

Erlotinib (ERL) is a tyrosine kinase inhibitor of epidermal growth factor receptor [15], [16]. As accumulating evidences suggest that over-expression of epidermal growth factor receptor relates to poor prognosis of pancreatic cancer [17]–[20]_ENREF_17, erlotinib has been considered as promising for treating APC in recent years. Moore et al [21] firstly demonstrated significantly improved outcomes by GEM/ERL combination therapy as compared with GEM alone in their study in 2007. After that, ERL was approved by US FDA for the treatment of APC. Later on, more studies [22], [23] were carried out to examine the efficacy of the GEM/ERL regimen. While the objective response rates, overall survivals, etc. differed in a wide range. The rates and severity of adverse events also varied greatly among studies. Notably, some extremely severe adverse events such as treatment-related death [21], [22], [24], [25] and gastrointestinal perforation [26] were reported.

As far as we know, there is lacking of a comprehensive summary on these issues. Therefore, we conducted this systematic review of the currently available studies (with or without comparison with GEM alone) to obtain a full view of the efficacy and safety profile of GEM/ERL for treating APC.

## Materials and Methods

### Literature Search

PubMed, EMBASE and The Cochrane Library were systematically searched in March 2012, without restrictions on language, to identify relevant publications. The detailed search strategy was described in Appendix S1. Briefly, both the MeSH terms and various text words for ‘pancreatic cancer’ were used in combination with those for ‘erlotinib’. The literature search was limited to “human studies”. We also reviewed recent conference abstracts of American Society of Clinical Oncology and European Society of Medical Oncology to identify ‘grey literature’. All potentially relevant studies were retrieved and their references were checked to see if there were additional eligible studies.

### Study Selection

To summarize the efficacy and safety profile of GEM/ERL therapy, studies meeting all of the following criteria were considered eligible to be included in the present review: 1) patients: APC; 2) treatment: GEM/ERL at any line; 3) outcomes: one or more of the following: objective response rate (the sum of complete response and partial response), disease control rate (the sum of complete response, partial response and stable disease), progression-free survival, time-to-progression, overall survival, 1-year survival rate and adverse events; 4) study design: single-arm retrospective or prospective study, which means that the GEM/ERL-treated arm of a randomized controlled trial could also be eligible. For the comparison of GEM/ERL with GEM alone, only randomized controlled trials were included, with the abovementioned criteria 1∼3 remaining unchanged.

When the same patient population was studied in more than one publication, only the one with most relevant data was included in this review. Two investigators independently reviewed the “potentially eligible” studies and then cross-checked their results. Disagreements between them were resolved by discussion. Unsettled disagreements were referred to a third expert for final decision.

### Data Extraction

The following data were extracted from included studies independently by two investigators: first author’s name, year of publication, study design, number of patients treated by GEM/ERL, age (median and range) of patients, percentage of male patients, performance status of patients, regimen of GEM/ERL, line of treatment, number and rate of objective response, number and rate of disease control, progression-free survival, time-to-progression, overall survival, 1-year survival rate, number and rate of each type of adverse events stratified by severity, and hazard ratio for the comparison of progression-free survival, time-to-progression or overall survival of GEM/ERL-treated patients with that of patients receiving GEM alone. We also extracted the results about potential predictive factors for the outcomes of GEM/ERL treatment from randomized controlled trials that assessed the treatment effect of GEM/ERL and conducted subgroup analyses according to different statuses or levels of the suspected factors, which allows the test for treatment-by-factor interaction.

### Statistical Analysis

The objective response rates, disease control rates and 1-year survival rates, respectively, were meta-analyzed by using the Meta-Analyst software with the random-effects model [27], [28]. The statistical heterogeneity among studies was assessed by the Cochran’s *Q*-test and the *I^2^* statistic [27], [29]. A *P* value ≤0.10 for the *Q*-test or an *I^2^*>50% was suggestive of substantial between-study heterogeneity. Subgroup analyses according to the dosage of GEM/ERL and study design features were conducted to explore the potential source of the heterogeneity. The progression-free survival, time-to-progression and overall survival with GEM/ERL, the comparison of them with their counterparts in patients treated with GEM alone and the results about potential predictive factors for GEM/ERL treatment were summarized descriptively due to inappropriateness for meta-analysis (see below). The adverse events rates from different studies were combined by simply summing up the events and totals, respectively, to produce rough estimates of the overall rates. Egger’s funnel plots [30] were initially planned to be employed but eventually not used to assess the possibility of publication bias, due to either the limited number of studies included for meta-analysis or the significant heterogeneity among studies [31]. No protocol of the present review has been published or registered.

## Results

### Literature Search and Study Characteristics

**Figure 1:**
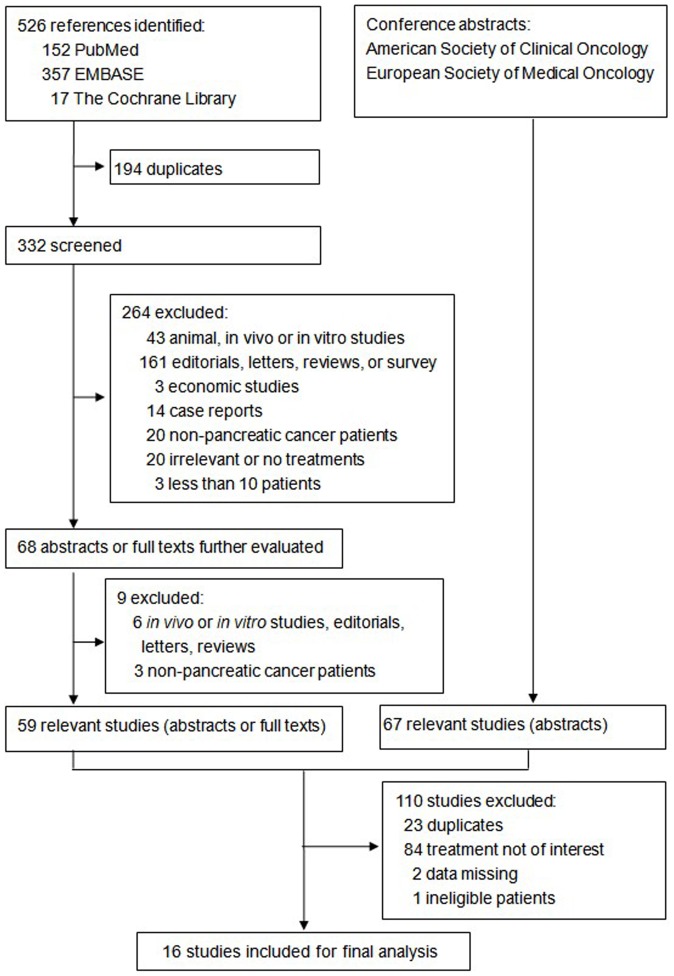
Flow chart of study selection.

The flow of study selection is demonstrated in Figure 1. Initially, 526 references were identified from PubMed, EMBASE and The Cochrane Library; 67 relevant conference abstracts were obtained by hand search of the websites of American Society of Clinical Oncology and European Society for Medical Oncology. After careful selection, a total of 16 eligible studies with 1,308 patients were included for our analysis.

Table 1 summarizes the basic characteristics of the included studies. Five of them were the GEM/ERL-treated arms of randomized controlled trials, one trial comparing GEM/ERL with GEM alone. In addition, there are nine single-arm trials and two retrospective patient series. The sample sizes of included studies ranged from 15 to 301. Most studies were conducted in Europeans and North Americans, only two in Asian populations (Japanese and Chinese). The median ages of patients in these studies were similar, which were around 61 to 66 years. Male accounted for 48%–65% of all subjects. Patients’ performance status before treatment was either over 60% by Karnofsky performance score or less than 2 by Eastern Cooperative Oncology Group performance score.

**Table 1:** Basic characteristic of included studies.

Study	Country	Design ofparent study	*N*	Median age (range)	Male	Performance status	Line	Criteria for response	Criteria for AEs	Treatment regimen
Cheng 2010^23^	China	Retrospective patient series	20	65 (36–77)	60%	KFS ≥60%	1^st^	RECIST	NCI CTC	GEM 1000 mg/m^2^, weekly × 2 every 3 weeks; ERL 100–150 mg/d
Aranda 2012^22^	Spain	Single–arm,phase II trial	153	64 (54–72)	54%	KFS ≥80%	1^st^	RECIST	NCI CTC	1000 mg/m^2^ of GEM, 30-min i.v. infusion, weekly × 3 every 4 weeks; ERL 100 mg/d
Ardavanis 2009^32^	Greece	Single-arm,phase II trial	27	63 (47–74)	59%	ECOG 0–2	1^st^	RECIST	NCI CTC	GEM 2000 mg/m^2^, 90-min i.v. infusion, in 2-week cycles; ERL 150 mg/d
Bengala 2009^33^	Italy	Single-arm,phase I/II trial	20	64 (50–79)	NA	NA	NA	NA	NA	GEM 1500–2500 mg/m^2^, 10 mg/m^2^/min, weekly × 2 every 4 weeks; ERL 100 mg/d
Milella 2010^37^	Italy	Single-arm,phase II trial	46	64 (35–81)	54%	NA	1^st^	RECIST	NA	GEM 1000 mg/m^2^, 10 mg/m^2^/min weekly; ERL 150 mg/d
Okusaka 2010^34^	Japan	Single-arm,phase II trial	106	62 (36–78)	53%	ECOG 0–1	1^st^	RECIST	NCI CTC	GEM 1000 mg/m^2^ weekly × 3 every 4 weeks; ERL 100 mg/d on days 3–28
Munoz Llarena 2011^24^	Spain	Single-arm,phase II trial	62	63 (37–78)	58%	ECOG 0–2	1^st^	NA	NA	GEM 1500 mg/m^2^, 10 mg/m^2^/min weekly × 3 every 4 weeks; ERL100 mg/d
Feliu 2011^6^	Spain	Single-arm,phase II trial	42	62 (47–79)	52%	ECOG 0–2	1^st^	RECIST	NCI CTC	GEM 1200 mg/m^2^, 120-min, weekly × 3 every 4 weeks; ERL 100 mg/d
Dragovich 2007^35^	US	Phase IB trial	15	66 (45–82)	50%	KPS ≥80%	NA	RECIST	NCI CTC	GEM 1000 mg/m^2^ weekly for 1st cycle (7 weeks), then weekly × 3 every 4 weeks; ERL 100 or 150 mg/d
Moore 2007^21^	Canada	Phase III RCT	285	64 (38–84)	48%	ECOG 0–2	1^st^	RECIST	NCI CTC	GEM 1000 mg/m^2^, 30-min, weekly × 7 for 8 weeks, then weekly × 3 every 4 weeks; ERL 100 or 150 mg/day
Boeck 2010^40^	Germany	Phase III RCT	58	63 (38–75)	57%	KPS ≥60%	1^st^	RECIST	NCI CTC	GEM 1000 mg/m^2^ weekly × 7 for 8 weeks, then weekly × 3 every 4 weeks; ERL 150 mg/d
Stuebs 2010^36^	Germany	Retrospective patient series	26	NA	NA	NA	1^st^	NA	NCI CTC	GEM 1000 mg/m^2^; ERL 100 mg/d
Modiano 2012^39^	US	Phase II RCT	39	NA	NA	NA		NA	NA	GEM NA; ERL 100 mg/d
Van Cutsem 2009^26^	Europe& US	Phase III RCT	301	61 (33–85)	62%	KPS ≥60%	1^st^	RECIST	NCI CTC	GEM 1000 mg/m^2^ weekly × 7 for 8 weeks, then weekly × 3 every 4 weeks; ERL 100 mg/d
Philip 2012^25^	US	Phase I/phaseII RCT	62	63	59%	NA	1^st^	NA	NCI CTC	GEM 1000 mg/m^2^ i.v. weekly × 3 for 4 weeks; ERL 100 mg/d
Kim 2011^38^	US	Phase II RCT	46	61	65%	ECOG 0–1	1^st^	NA	NA	GEM 1000 mg/m^2^ weekly × 4 every 4 week; ERL 100 mg/d

N, sample size; AE, adverse event; KFS, Karnofsky performance score; RECIST, Response Evaluation Criteria In Solid Tumors; NCI CTC, National Cancer Institute Common Terminology Criteria; GEM, gemcitabine; ERL, erlotinib; NA, not available; ECOG, Eastern Cooperative Oncology Group; RCT, randomized controlled trial.

GEM/ERL were given as first-line therapy to APC in all studies that reported relevant information. ERL was administered at the recommended dosage of 100 mg/day in 10 studies, 100–150 mg/day in three studies and 150 mg/day in the remaining three ones. Dosages of GEM varied from the mostly used and recommended 1000 mg/m^2^ to the investigational 2500 mg/m^2^. “Weekly for the first 3 weeks in a 4-week cycle” is the relatively common mode of administration interval. Tumor responses and the grades of adverse events were evaluated according to the Response Evaluation Criteria In Solid Tumors and National Cancer Institute Common Terminology Criteria, respectively, in all studies that specified these issues.

### Objective Response Rate

**Figure 2:**
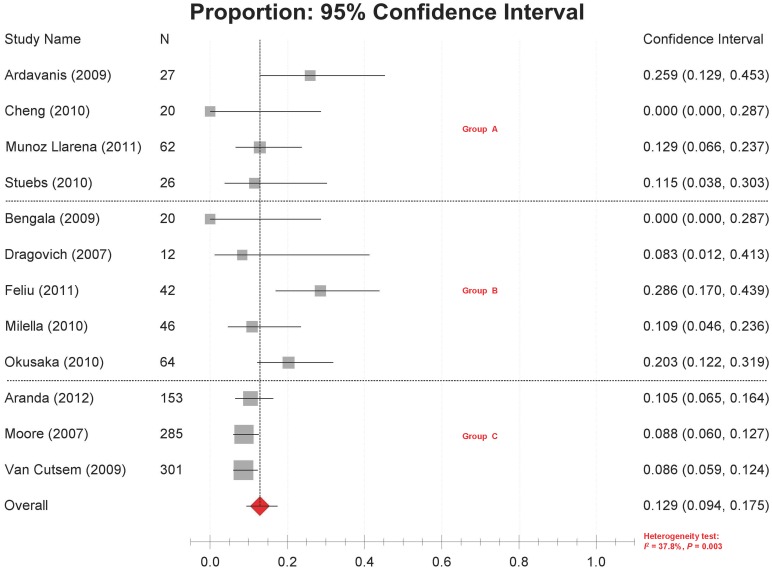
Forest plot of meta-analysis on objective response rates. Legends: Group A: retrospective small studies; Group B: prospective small studies; Group C: prospective large studies.

Objective response rates were reported in 12 studies [6], [21]–[24], [26], [32]–[37], ranging from 0% to 28.6% with significant between-study heterogeneity (*P* = 0.003, *I* ^2^ = 37.8%). The combined objective response rate estimated by the random-effects model was 12.9% (95% CI 9.4%–17.5%) (Figure 2).

Subgroup analyses were conducted to explore the potential sources of heterogeneity according to the GEM/ERL dosage, study design and sample sizes. As a result, the heterogeneity sustained within most subgroups (Table 2). In the ERL 100 mg/day plus GEM 1000 mg/m^2^ group (i.e. the recommended dosages for the combination use of them), the objective response rates ranged from 8.6% to 25.9% [26], [32], [34], [36]. Interestingly, the heterogeneity was significantly reduced within the subgroups defined by study design and sample size, i.e. retrospective small studies, prospective small studies and prospective large studies. The combined objective response rates for the three subgroups were 14.8%, 16.9% and 9.1%, respectively (Figure 2, Table 2).

**Table 2:** Results of subgroup analyses for objective response rate and disease control rate.

Outcomes and subgroups	No. of studies	Weighted estimates (%)	Heterogeneity test
**Objective response rate**			
1. Dosage of gemcitabine			
1000 mg/m2	8	10.5 (8.1–13.5)	*I* ^2^ = 20.8%, *P* = 0.180
>1000 mg/m2	4	19.3 (10.4–32.9)	*I* ^2^ = 36.3%, *P* = 0.067
2. Dosage of erlotinib			
100 mg/d	7	13.6 (8.9–20.2)	*I* ^2^ = 40.5%, *P* = 0.005
>100 mg/d	5	11.6 (6.3–20.2)	*I* ^2^ = 34.3%, *P* = 0.073
3. Combination			
Gemcitabine 1000 mg/m2+ erlotinib 100 mg/d	4	8.6 (5.9–12.4)	*I* ^2^ = 42.6%, *P* = 0.008
Other	8	13.3 (7.9–21.4)	*I* ^2^ = 39.5%, *P* = 0.005
4. Study design and sample size			
Retrospective small studies	4	14.8 (8.0–25.7)	*I* ^2^ = 27.1%, *P* = 0.159
Prospective small studies	5	16.9 (9.7–27.8)	*I* ^2^ = 32.6%, *P* = 0.092
Prospective large studies	3	9.1 (7.2–11.4)	*I* ^2^ = 0%, *P* = 0.443
**Disease control rate**			
1. Dosage of gemcitabine			
1000 mg/m2	8	55.3 (50.8–59.8)	*I* ^2^ = 23.9%, *P* = 0.151
>1000 mg/m2	4	53.7 (37.8–68.9)	*I* ^2^ = 41.3%, *P* = 0.017
2. Dosage of erlotinib			
100 mg/d	7	52.5 (44.9–55.9)	*I* ^2^ = 39.1%, *P* = 0.010
>100 mg/d	5	58.0 (53.0–62.8)	*I* ^2^ = 0%, *P* = 0.364
3. Combination			
Gemcitabine 1000 mg/m2+ erlotinib 100 mg/d	4	52.5 (45.3–59.7)	*I* ^2^ = 34.9%, *P* = 0.083
Other	8	57.4 (49.9–64.6)	*I* ^2^ = 31.7%, *P* = 0.066
4. Study design and sample size			
Retrospective small studies	4	55.3 (40.6–69.2)	*I* ^2^ = 38.2%, *P* = 0.046
Prospective small studies	5	52.3 (43.6–60.8)	*I* ^2^ = 32.7%, *P* = 0.077
Prospective large studies	3	57.0 (53.4–60.5)	*I* ^2^ = 0%, *P* = 0.340

### Disease Control Rate

**Figure 3:**
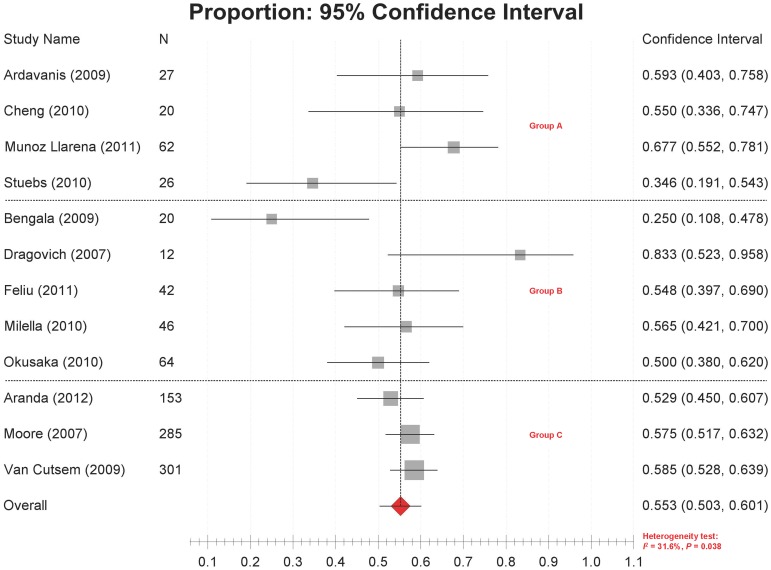
Forest plot of meta-analysis on disease control rates. Legends: Group A: retrospective small studies; Group B: prospective small studies; Group C: prospective large studies.

Disease control rates were reported in 12 studies [6], [21]–[24], [26], [32]–[37], ranging from 25.0% to 83.3%. Significant heterogeneity was detected among studies (*P* = 0.038, *I^2^* = 31.6%). The combined disease control rate was 55.3% (95% CI 50.3%–60.1%) (Figure 3).

The results of subgroup analyses (Table 2) indicated that the between-study heterogeneity cannot be satisfactorily explained by dosages or methodological characteristics. In the ERL 100 mg/day plus GEM 1000 mg/m^2^ group, the disease control rates ranged from 34.6% to 58.5%. Again, however, the results produced by large prospective studies were quite consistent, with a combined disease control rate of 57.0% (95% CI 53.4%–60.5%; heterogeneity test: *P* = 0.340, *I* ^2^ = 0,) (Figure 2, Table 2).

### Progression-Free Survival or Time-To-Progression

Progression-free survivals were reported in 10 studies [21]–[26], [34], [35], [37], [38], the median of which ranged from 2.0 to 9.6 months (mostly less than 5 months). Time-to-progression was reported in two studies [6], [32], the median values being 5 and 5.5 months, respectively.

### Survival

**Figure 4:**
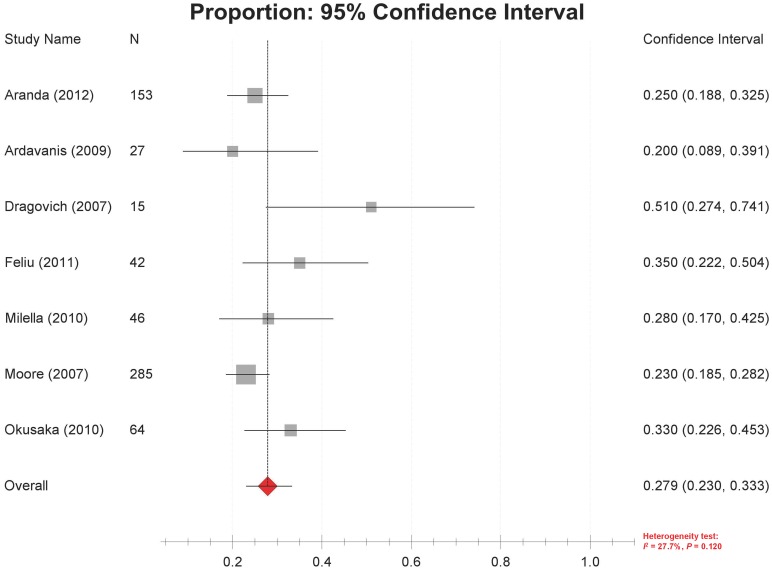
Forest plot of meta-analysis on 1-year survival rates.

Thirteen studies [6], [21]–[26], [32], [34]–[37], [39] reported data on the overall survival of GEM/ERL-treated patients, with a median of 5 to 12.5 months. One study reported a 6–month survival rate of 53% [37]. One-year survival rates were reported in seven studies [6], [21], [22], [32], [34], [35], [37], ranging from 20% to 51% (mostly below 35%), with no significant heterogeneity among them (*P* = 0.120, I^2^ = 27.7%). The combined rate by random-effect model was 27.9% (95% CI 23.0%–33.3%) (Figure 4).

### Toxicity

Data on the adverse events of GEM/ERL treatment were available in 14 studies [6], [21]–[26], [32]–[34], [36]–[38], [40]. In total, 45 types of adverse events involving different systems of the human body were reported (for full details, see Appendix S2). Three studies reported the total adverse event rates [21], [22], [36], which were about 96.3% (Table 3). The incidence of severe adverse events could be as high as 62.9%. The most commonly reported (defined by the number of studies with relevant data) adverse events were hematologic (anaemia [30.6%], leucopenia [71.2%], neutropenia [32.9%], thrombocytopenia [32.4%]), dermatologic (rash [57.9%]), gastrointestinal (diarrhea [47.0%], vomiting [35.8%], stomatitis [24.8%]), hepatobiliary (drug-induced liver injury) and some non-specific syndromes such as asthenia/fatigue (33.7%) and fever (29.0%) (Table 3). Other severe or clinically significant adverse events included treatment-related deaths (2.1%), gastrointestinal perforation (1.4%) and interstitial lung disease-like syndrome (2.5%), although they were low in incidence and were reported by few studies.

**Table 3:** Selected adverse events of gemcitabine/erlotinib treatment for advanced pancreatic cancer.

Type	No. of studies	Grade½ (%, n/*N*)	Grade¾ (%, n/*N*)	Total (%, n/*N*)
**All together**	3	–	62.9% (194/308)	96.3% (419/435)
**Treatment-related deaths**	4	–	–	2.1% (12/559)
**Hematologic**				
Anaemia	7	29.1% (164/563)	9.2% (62/671)	30.6% (156/509)
Leukocytopenia	4	60.9% (131/215)	18.6% (40/215)	71.2% (109/153)
Neutropenia	8	14.8% (94/635)	20.2% (150/743)	32.9% (209/635)
Thrombocytopenia	9	30.3% (204/673)	7.3% (57/781)	32.4% (206/635)
**Gastrointestinal/hepatobiliary**				
Anorexia	3	28.2% (154/546)	4.8% (26/546)	33.0% (180/546)
Diarrhea	11	44.8% (460/1026)	5.6% (59/1052)	47.0% (470/999)
Gastrointestinal perforations	1	0% (0/287)	1.4% (4/287)	1.4% (4/287)
Nausea	4	54.2% (247/456)	4.1% (20/482)	51.4% (202/393)
Stomatitis	5	26.5% (155/584)	1.2% (7/584)	24.8% (139/561)
Vomiting	4	37.5% (152/405)	4.0% (16/405)	35.8% (132/369)
**Dermatologic**				
Rash	11	40.5% (420/1038)	18.9% (208/1100)	57.9% (586/1012)
**Other**				
Asthenia/Fatigue	5	30.2% (232/768)	3.5% (27/768)	33.7% (259/768)
Fever	4	27.9% (160/573)	1.0% (6/573)	29.0% (166/573)
Hemarrhage	1	17.8% (51/287)	5.6% (16/287)	23.3% (67/287)
Infection	3	27.3% (105/385)	19.5% (75/385)	40.4% (139/344)
ILD-like syndrome	1	–	–	2.5% (7/282)

N, total number of patients; ILD, interstitial lung disease.

### Comparison of GEM/ERL with GEM Alone

Among the randomized controlled trials we identified, only Moore et al [21] examined the benefit introduced by the addition of ERL to GEM. The objective response rates in GEM/ERL and GEM/placebo arms were 8.6% and 8.0%, respectively, and disease control rates were 57.5% and 49.2%, respectively, both differences statistically insignificant. However, progression-free survival (3.75 vs. 3.55 months, HR = 0.77, 95% CI 0.64–0.92), overall survival (6.24 vs. 5.91 months, HR = 0.82, 95% CI 0.69–0.99) and 1-year survival rate (23% vs. 17%) were all significantly better in GEM/ERL arm than in GEM/placebo arm. As for toxicities, patients treated with GEM/ERL had higher frequencies of rash, diarrhea, infection and stomatitis, although all of these were at Grade 1/2. Moreover, interstitial lung disease-like syndrome (7 vs. 1) and protocol-related deaths (6 vs. 0) were much more in the GEM/ERL arm than in GEM/placebo arm.

### Factors Predictive of the Outcomes of GEM/ERL Treatment

Two randomized controlled trials [21], [26] evaluating the effect of GEM/ERL tried to identify potential predictive factors for efficacy of the treatment by conducting subgroup analyses according to different statuses or levels of these factors. Moore et al [21] found that in patients with one of the following characteristics GEM/ERL treatment significantly improved the overall survival as compared with GEM alone, whereas in patients without the characteristics the treatment efficacy was not significant: age ≤65 (vs >65), Eastern Cooperative Oncology Group performance score = 2 (vs 0 or 1), male (vs female), pain score ≤20 (vs >20) and distant metastatic (vs locally advanced). The treatment efficacy did not differ according to the expression status of epidermal growth factor receptor. Van Custem et al [26] found that the efficacy of GEM/ERL in terms of overall survival was significantly worse than bevacizumab in combination with GEM/ERL among patients with baseline C-reactive protein >1.4 (vs ≤1.4), baseline lactate dehydrogenase>upper limit of normal (vs ≤ upper limit of normal) or primary tumor located in pancreas tail (vs body or head), but did not differ much according to sex, age, race, Karnofsky performance score and other laboratory indexes.

## Discussion

The present systematic review provides a comprehensive overview of current evidences on the efficacy and safety and GEM/ERL treatment for APC. Although there existed significant heterogeneity in the overall meta-analyses of objective response rate and disease control rate, results from large prospective studies were consistent, which strengthened the robustness of our conclusion.

GEM was once used as a monotherapy for APC. As reported by previous studies, the objective response rate of patients treated by GEM alone varied from 4.4% to 23.8%, and the disease control rate could be up to 48.4% [8], [10], [12]. The progression-free survival or time-to-progression with GEM is about 2.2 to 4 months, mainly around 3 to 4 months, and the corresponding overall survival is about 5.4 to 8.2 months, mostly 5.4 to 7 months, with a 1-year survival rate of 18% to 37.2% [8], [9], [41].

According to this systematic review, the objective response rate in GEM/ERL-treated APC patients varied from 0 to 28.6%, with a weighted estimate of 9.1% based on three large prospective studies. The disease control rate reported by existing studies ranged from 25% to 71%, mostly around 50%–60%, with a weighted estimate of 55.3%, which is consistent with the results from the three large prospective studies. The progression-free survival or time-to-progression after GEM/ERL is about 2.0 to 9.6 months, mostly 3–5 months, and the overall survival varied from 5 to 12.5 months, with the 1-year survival rates ranging from 20%–51% (weighted mean 27.9%).

Based on the above data, it seems that the objective response rate and disease control rate with GEM/ERL and those with GEM alone are not much different from each other. This is consistent with the study of Moore et al which directly compared GEM/ERL with GEM alone and found no significant differences in terms of objective response rate and disease control rate. However, studies have shown that objective response rate was not a good predictor for the most important clinical outcome, i.e. survival. For example, the 1-year survival rate of patients with a relatively low objective response rate could be fairly high [42], [43].

According to the ranges reported by existing studies as mentioned above, the progression-free survival/time-to-progression and overall survival with GEM/ERL seemed to be slightly better than those with GEM monotherapy. This is also consistent with the results from the direct comparison of GEM/ERL with GEM alone conducted by Moore et al, which showed that the progression-free survival and overall survival after GEM/ERL treatment were both statistically significantly longer than those with GEM alone [21], although the absolute improvements in median progression-free survival and overall survival were only about 0.2 and 0.3 months, respectively. Similarly, the 1-year survival rate of patients treated by GEM/ERL was slightly, but statistically significantly higher than that after GEM monotherapy [21].

For clinicians and patients faced with options of GEM/ERL or GEM in combination with other chemotherapy drugs, it would be of interest to know whether the two kinds of treatments are equally effective and safe. According to published studies that have investigated the efficacy of GEM plus other chemotherapies, the objective response rates varied from 6.9% to 26.8%, disease control rates from 46% to 70.4%, overall survivals from 6.3 to 9.5 months and 1-year survival rates from 21.4%–34.8%, all of which seemed to be similar with the efficacy of GEM/ERL [9]–[12], [41], [44], [45]. The progression-free survival with GEM plus other chemotherapies ranged from 3.4 to 5.8 months [9], [11], seemingly slightly better than that with GEM/ERL. However, few studies have directly compared the two kinds of treatment in a single trial. A retrospective multivariate matched pair analysis conducted by Stuebs et al compared GEM/ERL with GEM in combination with docetaxel for the treatment of APC [36]. They observed no advantages of one treatment over the other and concluded that “both treatment options can be applied safely and effectively with a moderate toxicity profile as first-line treatment”.

The adverse events rate of GEM/ERL is quite high, which not surprising in the realm of cancer chemotherapy. The only one randomized controlled trial [21] comparing GEM/ERL with GEM alone found that there were more treatment-related deaths and interstitial lung disease-like syndrome in the GEM/ERL arm (although the incidence rate is low), warranting close monitoring. Interestingly, some studies have found that the severity of some adverse events were associated with prognosis. For example, Moore et al and Aranda et al prospectively confirmed that patients with grade ≥2 rash had significantly longer progression-free survival and overall survival than did those with grade <2 rash; the objective response rate and disease control rate were also much higher in patients with grade ≥2 rash [21], [22]. This indicated that the presence of adverse events may not always be a bad thing.

The present systematic review has some limitations. First, full-text papers could not be identified for 7 (44%) of the included studies [24], [25], [33], [36]–[39], which precluded us from obtaining more complete information on the treatment regimens and clinical outcomes and from conducting more in-depth analysis. Second, we did not conduct weighted meta-analysis of the results on adverse events. Nevertheless, we believe that our overall conclusions would not be affected much by the two issues, considering the relatively large dataset we have compiled and the consistent results from large prospective studies. The third limitation has to do with the potential predictive factors for GEM/ERL treatment. Although it was found that some factors might predict the efficacy of GEM/ERL treatment, evidences on this issue have been scarce, inconsistent and were based on subgroup analyses that did not deal with the possibility of “false positive” raised by multiple testing. In addition, socioeconomic factors, nutrition and mental statuses of patients might also influence the outcomes of GEM/ERL treatment; however, due to lack of relevant data from the included original studies, we were unable to examine their roles which might be addressed by future studies.

In conclusion, GEM/ERL represent a new option for treating APC, with mild but clinically meaningful additive efficacy compared with GEM alone. The related adverse events are generally acceptable, although careful management is needed for several important and specific adverse events. Prognostic and predictive factors for both efficacy and toxicity outcomes may be warranted to be further studied.

## Supporting Information

Appendix S1 **Detailed search strategy.**
(DOCX)

Appendix S2 **All reported adverse events of gemcitabine/erlotinib treatment for advanced pancreatic cancer.**
(DOCX)

## References

[1] Bond-Smith G, Banga N, Hammond TM, Imber CJ (2012) Pancreatic adenocarcinoma. BMJ 344: e2476. 22592847 10.1136/bmj.e2476

[2] Lowenfels AB, Maisonneuve P (2006) Epidemiology and risk factors for pancreatic cancer. Best Pract Res Clin Gastroenterol 20: 197–209. 16549324 10.1016/j.bpg.2005.10.001

[3] Anderson K MT, Silverman DT (2006) Cancer of the pancreas. In: Schottenfeld D, Fraumeni JF Jr, eds. Cancer epidemiology and prevention. 3rd ed. Oxford University Press.

[4] Jemal A, Siegel R, Ward E, Hao Y, Xu J, et al (2008) Cancer statistics, 2008. CA Cancer J Clin 58: 71–96. 18287387 10.3322/CA.2007.0010

[5] Ferlay J, Autier P, Boniol M, Heanue M, Colombet M, et al (2007) Estimates of the cancer incidence and mortality in Europe in 2006. Ann Oncol 18: 581–592. 17287242 10.1093/annonc/mdl498

[6] Feliu J, Borrega P, Leon A, Lopez-Gomez L, Lopez M, et al (2011) Phase II study of a fixed dose-rate infusion of gemcitabine associated with erlotinib in advanced pancreatic cancer. Cancer Chemother Pharmacol 67: 215–221. 20927525 10.1007/s00280-010-1472-0

[7] Cascinu S, Graziano F, Catalano G (1999) Chemotherapy for advanced pancreatic cancer: it may no longer be ignored. Ann Oncol 10: 105–109. 10.1023/a:1008205515591 10076729

[8] Burris HA 3rd, Moore MJ, Andersen J, Green MR, Rothenberg ML, et al (1997) Improvements in survival and clinical benefit with gemcitabine as first-line therapy for patients with advanced pancreas cancer: a randomized trial. J Clin Oncol 15: 2403–2413. 9196156 10.1200/JCO.1997.15.6.2403

[9] Berlin JD, Catalano P, Thomas JP, Kugler JW, Haller DG, et al (2002) Phase III study of gemcitabine in combination with fluorouracil versus gemcitabine alone in patients with advanced pancreatic carcinoma: Eastern Cooperative Oncology Group Trial E2297. J Clin Oncol 20: 3270–3275. 12149301 10.1200/JCO.2002.11.149

[10] Heinemann V, Quietzsch D, Gieseler F, Gonnermann M, Schonekas H, et al (2006) Randomized phase III trial of gemcitabine plus cisplatin compared with gemcitabine alone in advanced pancreatic cancer. J Clin Oncol 24: 3946–3952. 16921047 10.1200/JCO.2005.05.1490

[11] Louvet C, Labianca R, Hammel P, Lledo G, Zampino MG, et al (2005) Gemcitabine in combination with oxaliplatin compared with gemcitabine alone in locally advanced or metastatic pancreatic cancer: results of a GERCOR and GISCAD phase III trial. J Clin Oncol 23: 3509–3516. 15908661 10.1200/JCO.2005.06.023

[12] Rocha Lima CM, Green MR, Rotche R, Miller WH Jr, Jeffrey GM, et al (2004) Irinotecan plus gemcitabine results in no survival advantage compared with gemcitabine monotherapy in patients with locally advanced or metastatic pancreatic cancer despite increased tumor response rate. J Clin Oncol 22: 3776–3783. 15365074 10.1200/JCO.2004.12.082

[13] Colucci G, Giuliani F, Gebbia V, Biglietto M, Rabitti P, et al (2002) Gemcitabine alone or with cisplatin for the treatment of patients with locally advanced and/or metastatic pancreatic carcinoma: a prospective, randomized phase III study of the Gruppo Oncologia dell’Italia Meridionale. Cancer 94: 902–910. 11920457

[14] Miller VA, Kris MG (2000) Docetaxel (Taxotere) as a single agent and in combination chemotherapy for the treatment of patients with advanced non-small cell lung cancer. Semin Oncol 27: 3–10. 10810932

[15] Lynch TJ Jr, Kim ES, Eaby B, Garey J, West DP, et al (2007) Epidermal growth factor receptor inhibitor-associated cutaneous toxicities: an evolving paradigm in clinical management. Oncologist 12: 610–621. 17522250 10.1634/theoncologist.12-5-610

[16] Perez-Soler R, Saltz L (2005) Cutaneous adverse effects with HER1/EGFR-targeted agents: is there a silver lining? J Clin Oncol 23: 5235–5246. 16051966 10.1200/JCO.2005.00.6916

[17] Tobita K, Kijima H, Dowaki S, Kashiwagi H, Ohtani Y, et al (2003) Epidermal growth factor receptor expression in human pancreatic cancer: Significance for liver metastasis. Int J Mol Med 11: 305–309. 12579331

[18] Fjallskog ML, Lejonklou MH, Oberg KE, Eriksson BK, Janson ET (2003) Expression of molecular targets for tyrosine kinase receptor antagonists in malignant endocrine pancreatic tumors. Clin Cancer Res 9: 1469–1473. 12684421

[19] Xiong HQ (2004) Molecular targeting therapy for pancreatic cancer. Cancer Chemother Pharmacol 54 Suppl 1 S69–77. 15316751 10.1007/s00280-004-0890-2

[20] Ueda S, Ogata S, Tsuda H, Kawarabayashi N, Kimura M, et al (2004) The correlation between cytoplasmic overexpression of epidermal growth factor receptor and tumor aggressiveness: poor prognosis in patients with pancreatic ductal adenocarcinoma. Pancreas 29: e1–8. 15211117 10.1097/00006676-200407000-00061

[21] Moore MJ, Goldstein D, Hamm J, Figer A, Hecht JR, et al (2007) Erlotinib plus gemcitabine compared with gemcitabine alone in patients with advanced pancreatic cancer: a phase III trial of the National Cancer Institute of Canada Clinical Trials Group. J Clin Oncol 25: 1960–1966. 17452677 10.1200/JCO.2006.07.9525

[22] Aranda E, Manzano JL, Rivera F, Galan M, Valladares-Ayerbes M, et al (2012) Phase II open-label study of erlotinib in combination with gemcitabine in unresectable and/or metastatic adenocarcinoma of the pancreas: relationship between skin rash and survival (Pantar study). Ann Oncol 23: 1919–1925. 22156621 10.1093/annonc/mdr560

[23] Cheng YJ, Bai CM, Zhang ZJ (2010) Efficacy of gemcitabine combined with erlotinib in patients with advanced pancreatic cancer. Zhongguo Yi Xue Ke Xue Yuan Xue Bao 32: 421–423. 20868602 10.3881/j.issn.1000-503X.2010.04.013

[24] Munoz Llarena A, Mane J, Lopez-Vivanco G, Ruiz de Lobera A, Sancho A, et al. (2011) Gemcitabine (G) fixed-dose-rate infusion (FDR) plus erlotinib (E) in patients with advanced pancreatic cancer (APC). Available: http://www.asco.org/ASCOv2/Meetings/Abstracts?&vmview=abst_detail_view&confID=103&abstractID=71207. Accessed 7 November 2012.

[25] Philip PA, Goldman BH, Ramanathan RK, Lenz HJ, Lowy AM, et al. (2012) Phase I randomized phase II trial of gemcitabine, erlotinib, and cixutumumab versus gemcitabine plus erlotinib as first-line treatment in patients with metastatic pancreatic cancer (SWOG-0727). Available: http://www.asco.org/ASCOv2/Meetings/Abstracts?&vmview=abst_detail_view&confID=115&abstractID=87986. Accessed 7 November 2012.

[26] Van Cutsem E, Vervenne WL, Bennouna J, Humblet Y, Gill S, et al (2009) Phase III trial of bevacizumab in combination with gemcitabine and erlotinib in patients with metastatic pancreatic cancer. J Clin Oncol 27: 2231–2237. 19307500 10.1200/JCO.2008.20.0238

[27] Deeks JJ, Higgins JPT, Altman DG, editors. Analysing and presenting results. In: Higgins JPT, Green S, editors. Cochrane Handbook for Systematic Reviews of Interventions 4.2.6 [updated September 2006]; Section 8. In: The Cochrane Library, Issue 4, 2006. Chichester, UK: John Wiley & Sons, Ltd.

[28] DerSimonian R, Laird N (1986) Meta-analysis in clinical trials. Control Clin Trials 7: 177–188. 3802833 10.1016/0197-2456(86)90046-2

[29] Higgins JP, Thompson SG, Deeks JJ, Altman DG (2003) Measuring inconsistency in meta-analyses. Bmj 327: 557–560. 12958120 10.1136/bmj.327.7414.557 PMC192859

[30] Egger M, Davey Smith G, Schneider M, Minder C (1997) Bias in meta-analysis detected by a simple, graphical test. Bmj 315: 629–634. 9310563 10.1136/bmj.315.7109.629 PMC2127453

[31] Lau J, Ioannidis JP, Terrin N, Schmid CH, Olkin I (2006) The case of the misleading funnel plot. Bmj 333: 597–600. 16974018 10.1136/bmj.333.7568.597 PMC1570006

[32] Ardavanis A, Kountourakis P, Karagiannis A, Doufexis D, Tzovaras AA, et al (2009) Biweekly gemcitabine (GEM) in combination with erlotinib (ERL): an active and convenient regimen for advanced pancreatic cancer. Anticancer Res 29: 5211–5217. 20044638

[33] Bengala C, Sternieri R, Malavasi N, Ponti G, Bertolini F, et al. (2009) Phase II trial of erlotinib in combination with increasing dose of gemcitabine given as fixed dose rate infusion in advanced pancreatic cancer (APC). Available: http://www.asco.org/ASCOv2/Meetings/Abstracts?&vmview=abst_detail_view&confID=63&abstractID=10332. Accessed 7 November 2012.

[34] Okusaka T, Furuse J, Funakoshi A, Ioka T, Yamao K, et al (2010) Phase II study of erlotinib plus gemcitabine in Japanese patients with unresectable pancreatic cancer. Cancer Sci 102: 425–431. 21175992 10.1111/j.1349-7006.2010.01810.x

[35] Dragovich T, Huberman M, Von Hoff DD, Rowinsky EK, Nadler P, et al (2007) Erlotinib plus gemcitabine in patients with unresectable pancreatic cancer and other solid tumors: phase IB trial. Cancer Chemother Pharmacol 60: 295–303. 17149608 10.1007/s00280-006-0389-0

[36] Stuebs P, Habermann P, Zierau K, Schuette K, Fahlke J, et al. (2010) First-line therapy for advanced pancreatic cancer with gemcitabine and docetaxel versus gemcitabine and erlotinib: A multivariate matched pair analysis. Available: http://www.asco.org/ASCOv2/Meetings/Abstracts?&vmview=abst_detail_view&confID=74&abstractID=52331. Accessed: 7 November 2012.

[37] Milella M, Vaccaro V, Sperduti I, Bria E, Gelibter A, et al. (2010) Phase II study of erlotinib (E) combined with fixed dose-rate gemcitabine (FDR-Gem) as first-line treatment for advanced adenocarcinoma of the pancreas (PDAC). Available: http://www.asco.org/ASCOv2/Meetings/Abstracts?&vmview=abst_detail_view&confID=74&abstractID=51951. Accessed: 7 November 2012.

[38] Kim GP, Foster NR, Salim M, Flynn PJ, Moore DF, et al. (2011) Randomized phase II trial of panitumumab (P), erlotinib (E), and gemcitabine (G) versus erlotinib-gemcitabine in patients with untreated, metastatic pancreatic adenocarcinoma. Available: http://www.asco.org/ascov2/Meetings/Abstracts?&vmview=abst_detail_view&confID=102&abstractID=82619. Accessed: 7 November 2012.

[39] Modiano M, Keogh GP, Manges R, Stella PJ, Milne G, et al. (2012) Apricot-P: A randomized placebo-controlled phase II study of COX-2 inhibitor apricoxib or placebo in combination with gemcitabine and erlotinib in advanced or metastatic adenocarcinoma of the pancreas. Available: http://www.asco.org/ASCOv2/Meetings/Abstracts?&vmview=abst_detail_view&confID=115&abstractID=87740. Accessed: 7 November 2012.

[40] Boeck S, Vehling-Kaiser U, Waldschmidt D, Kettner E, Marten A, et al (2010) Erlotinib 150 mg daily plus chemotherapy in advanced pancreatic cancer: an interim safety analysis of a multicenter, randomized, cross-over phase III trial of the ‘Arbeitsgemeinschaft Internistische Onkologie’. Anticancer Drugs 21: 94–100. 19770635 10.1097/CAD.0b013e32833123ed

[41] Scheithauer W, Schull B, Ulrich-Pur H, Schmid K, Raderer M, et al (2003) Biweekly high-dose gemcitabine alone or in combination with capecitabine in patients with metastatic pancreatic adenocarcinoma: a randomized phase II trial. Ann Oncol 14: 97–104. 12488300 10.1093/annonc/mdg029

[42] Androulakis N, Kourousis C, Dimopoulos MA, Samelis G, Kakolyris S, et al (1999) Treatment of pancreatic cancer with docetaxel and granulocyte colony-stimulating factor: a multicenter phase II study. J Clin Oncol 17: 1779–1785. 10561215 10.1200/JCO.1999.17.6.1779

[43] Stathopoulos GP, Androulakis N, Souglakos J, Stathopoulos J, Georgoulias V (2008) Present treatment and future expectations in advanced pancreatic cancer. Anticancer Res 28: 1303–1308. 18505070

[44] Oettle H, Richards D, Ramanathan RK, van Laethem JL, Peeters M, et al (2005) A phase III trial of pemetrexed plus gemcitabine versus gemcitabine in patients with unresectable or metastatic pancreatic cancer. Ann Oncol 16: 1639–1645. 16087696 10.1093/annonc/mdi309

[45] Stathopoulos GP, Mavroudis D, Tsavaris N, Kouroussis C, Aravantinos G, et al (2001) Treatment of pancreatic cancer with a combination of docetaxel, gemcitabine and granulocyte colony-stimulating factor: a phase II study of the Greek Cooperative Group for Pancreatic Cancer. Ann Oncol 12: 101–103. 11249034 10.1023/a:1008310106171

