# Supplementary material for: Gemcitabine Plus Erlotinib for Advanced Pancreatic Cancer: A Systematic Review with Meta-Analysis
Source: PLoS One. 2013 Mar 5;8(3):e57528. doi: 10.1371/journal.pone.0057528 (PMC3589410; doi:10.1371/journal.pone.0057528)
Supplement: Appendix S1 — Detailed search strategy. (DOCX) [file pone.0057528.s001.docx]

**Appendix S1. Detailed search strategy**

**1. PubMed**

| Search | Query | Items found |
| --- | --- | --- |
| [#7](http://www.ncbi.nlm.nih.gov/pubmed/advanced) | Search **#3 NOT #6** | [152](http://www.ncbi.nlm.nih.gov/pubmed/?cmd=HistorySearch&querykey=11) |
| [#6](http://www.ncbi.nlm.nih.gov/pubmed/advanced) | Search **#4 OR #5** | [75](http://www.ncbi.nlm.nih.gov/pubmed/?cmd=HistorySearch&querykey=9) |
| [#5](http://www.ncbi.nlm.nih.gov/pubmed/advanced) | Search **#3** Limits: **Editorial, Letter, Case Reports, Comment, In Vitro** | [27](http://www.ncbi.nlm.nih.gov/pubmed/?cmd=HistorySearch&querykey=8) |
| [#4](http://www.ncbi.nlm.nih.gov/pubmed/advanced) | Search **#3** Limits: **Animals** | [49](http://www.ncbi.nlm.nih.gov/pubmed/?cmd=HistorySearch&querykey=7) |
| [#3](http://www.ncbi.nlm.nih.gov/pubmed/advanced) | Search **#1 AND #2** | [227](http://www.ncbi.nlm.nih.gov/pubmed/?cmd=HistorySearch&querykey=6) |
| [#2](http://www.ncbi.nlm.nih.gov/pubmed/advanced) | Search **(pancreas OR pancreatic) AND (cancer OR carcinoma)** Field: **Title/Abstract** | [34182](http://www.ncbi.nlm.nih.gov/pubmed/?cmd=HistorySearch&querykey=5) |
| [#1](http://www.ncbi.nlm.nih.gov/pubmed/advanced) | Search **erlotinib OR tarceva OR "CP 358774" OR "CP 358,774" OR CP-358774 OR "CP-358,774" OR "CP-358 774" OR OSI-774** Field: **Title/Abstract** | [2226](http://www.ncbi.nlm.nih.gov/pubmed/?cmd=HistorySearch&querykey=4) |

- [AND in builder](http://www.ncbi.nlm.nih.gov/pubmed/advanced)
- [OR in builder](http://www.ncbi.nlm.nih.gov/pubmed/advanced)
- [NOT in builder](http://www.ncbi.nlm.nih.gov/pubmed/advanced)
- [Delete from history](http://www.ncbi.nlm.nih.gov/pubmed/advanced)
- [Show search results](http://www.ncbi.nlm.nih.gov/pubmed/advanced)
- [Show search details](http://www.ncbi.nlm.nih.gov/pubmed/advanced)
- [AND in builder](http://www.ncbi.nlm.nih.gov/pubmed/advanced)
- [OR in builder](http://www.ncbi.nlm.nih.gov/pubmed/advanced)
- [NOT in builder](http://www.ncbi.nlm.nih.gov/pubmed/advanced)
- [Delete from history](http://www.ncbi.nlm.nih.gov/pubmed/advanced)
- [Show search results](http://www.ncbi.nlm.nih.gov/pubmed/advanced)
- [Show search details](http://www.ncbi.nlm.nih.gov/pubmed/advanced)
- [Save in My NCBI](http://www.ncbi.nlm.nih.gov/pubmed/advanced)
- [AND in builder](http://www.ncbi.nlm.nih.gov/pubmed/advanced)
- [OR in builder](http://www.ncbi.nlm.nih.gov/pubmed/advanced)
- [NOT in builder](http://www.ncbi.nlm.nih.gov/pubmed/advanced)
- [Show search results](http://www.ncbi.nlm.nih.gov/pubmed/advanced)
- [Save as a My NCBI Collection](http://www.ncbi.nlm.nih.gov/pubmed/advanced)

**2. EMBASE**

| **[# ▲](http://ovidsp.tx.ovid.com/sp-3.5.1a/ovidweb.cgi?&S=PJOMFPDPNLDDIKHLNCALMCGCEMHFAA00&Sort+Sets=descending)** | **Searches** | **Results** |
| --- | --- | --- |
| 1 | (erlotinib or tarceva or "CP 358774" or "CP 358,774" or CP-358774 or "CP-358,774" or "CP-358 774" or OSI-774).ti. | 1512 |
| 2 | (erlotinib or tarceva or "CP 358774" or "CP 358,774" or CP-358774 or "CP-358,774" or "CP-358 774" or OSI-774).ab. | 3137 |
| 3 | 1 or 2 | 3462 |
| 4 | ((pancreas or pancreatic) and (cancer or carcinoma)).ti. | 19514 |
| 5 | ((pancreas or pancreatic) and (cancer or carcinoma)).ab. | 35872 |
| 6 | 4 or 5 | 41058 |
| 7 | 3 and 6 | 357 |

**3. Cochrane Central Register of Controlled Trials (Trials)**

| **ID** | **Search** | **Hits** |
| --- | --- | --- |
| #1 | [(erlotinib OR tarceva OR "CP 358774" OR "CP 358,774" OR CP-358774 OR "CP-358,774" OR "CP-358 774" OR OSI-774):ti in Trials](http://onlinelibrary.wiley.com/o/cochrane/searchHistory?mode=runquery&qnum=1) | 94 |
| #2 | [(erlotinib OR tarceva OR "CP 358774" OR "CP 358,774" OR CP-358774 OR "CP-358,774" OR "CP-358 774" OR OSI-774):ab in Trials](http://onlinelibrary.wiley.com/o/cochrane/searchHistory?mode=runquery&qnum=2) | 59 |
| #3 | [(pancreas OR pancreatic) AND (cancer OR carcinoma):ti in Trials](http://onlinelibrary.wiley.com/o/cochrane/searchHistory?mode=runquery&qnum=3) | 542 |
| #4 | [(pancreas OR pancreatic) AND (cancer OR carcinoma):ab in Trials](http://onlinelibrary.wiley.com/o/cochrane/searchHistory?mode=runquery&qnum=4) | 565 |
| #5 | [(( #1 OR #2 ) AND ( #3 OR #4 ))](http://onlinelibrary.wiley.com/o/cochrane/searchHistory?mode=runquery&qnum=5) | 17 |
