# Supplementary material for: Gemcitabine Plus Erlotinib for Advanced Pancreatic Cancer: A Systematic Review with Meta-Analysis
Source: PLoS One. 2013 Mar 5;8(3):e57528. doi: 10.1371/journal.pone.0057528 (PMC3589410; doi:10.1371/journal.pone.0057528)
Supplement: Appendix S2 — All reported adverse events of gemcitabine/erlotinib treatment for advanced pancreatic cancer. (DOCX) [file pone.0057528.s002.docx]

Appendix S2. All reported adverse events of gemcitabine/erlotinib treatment for advanced pancreatic cancer

| **Type** | **Grade 1/2** | **Grade 3/4** | **Total** | **References** |
| --- | --- | --- | --- | --- |
| **Total adverse events** | - - | 62.9% (194/308) | 96.3% (419/435) | 1, 13, 10 |
| **Treatment-related deaths** | - - | - - | 2.1% (12/559) | 1, 8, 10, 12 |
| **Hematologic** | | | | |
| Anaemia | 29.1% (164/563) | 9.2% (62/671) | 30.6% (156/509) | 1, 2, 9, 8, 6, 4, 14 |
| Hemoglobin decreased | 57.5% (61/106) | 14.2% (15/106) | 71.7% (76/106) | 11 |
| Hematocrit decreased | 61.3% (65/106) | 7.5% (8/106) | 68.9% (73/106) | 11 |
| Hematologic toxicities | - - | 38.7% (24/62) | 70.0% (14/20) | 5, 12 |
| Leukocytopenia | 60.9% (131/215) | 18.6% (40/215) | 71.2% (109/153) | 5, 2, 11, 4 |
| Lymphocyte count decreased | 30.2% (32/106) | 13.2% (14/106) | 43.4% (46/106) | 11 |
| Neutropenia | 14.8% (94/635) | 20.2% (150/743) | 32.9% (209/635) | 1, 2, 3, 9, 11, 8, 6, 14 |
| Red blood cell count decreased | 60.4% (64/106) | 7.5% (8/106) | 67.9% (72/106) | 11 |
| Thrombocytopenia | 30.3% (204/673) | 7.3% (57/781) | 32.4% (206/635) | 5, 1, 2, 9, 11, 8, 6, 4, 14 |
| **Gastrointestinal/hepatobiliary** | | | | |
| Alkaline phosphatase | 29.6% (8/27) | 3.7% (1/27) | 33.3% (9/27) | 2 |
| Anorexia | 28.2% (154/546) | 4.8% (26/546) | 33.0% (180/546) | 1, 11, 14 |
| ALT increased | 36.5% (54/148) | 7.4% (11/148) | 43.9% (65/148) | 11, 6 |
| AST increased | 37.8% (56/148) | 4.1% (6/148) | 41.9% (62/148) | 11, 6 |
| Constipation | 19.9% (93/467) | 0.6% (3/467) | 20.6% (96/467) | 1, 2, 14 |
| Diarrhea | 44.8% (460/1026) | 5.6% (59/1052) | 47.0% (470/999) | 5, 1, 2, 3, 11, 8, 6, 13, 10, 4, 14 |
| Dysgeusia | 5.2% (8/153) | 0% (0/153) | 5.2% (8/153) | 1 |
| Elevated transaminases | 25% (5/20) | 7.6% (5/66) | 25% (5/20) | 5, 9 |
| Gama-glutamyltransferase increased | 20.8% (22/106) | 12.3% (13/106) | 33.0% (35/106) | 11 |
| Gastrointestinal perforations | 0% (0/287) | 1.4% (4/287) | 1.4% (4/287) | 14 |
| Mucositis | 8.1% (5/62) | 1.6% (1/62) | 9.7% (6/62) | 8 |
| Nausea | 54.2% (247/456) | 4.1% (20/482) | 51.4% (202/393) | 11, 13, 4, 14 |
| Serum transaminases | 25.9% (7/27) | 0% (0/27) | 25.9% (7/27) | 2, |
| Stomatitis | 26.5% (155/584) | 1.2% (7/584) | 24.8% (139/561) | 1, 3, 11, 10, 4 |
| Total bilirubin | 33.3% (9/27) | 3.7% (1/27) | 37.0% (10/27) | 2, |
| Vomiting | 37.5% (152/405) | 4.0% (16/405) | 35.8% (132/369) | 3, 8, 4, 14 |
| **Dermatologic** | | | | |
| Acne | - - | 15.4% (4/26) | - - | 13 |
| Alopecia | 25.9% (7/27) | 0% (0/27) | 25.9% (7/27) | 2 |
| Cutaneous | 81.5% (22/27) | 7.4% (2/27) | 88.9% (24/27) | 2 |
| Dry skin | 46.2% (49/106) | 0% (0/106) | 46.2% (49/106) | 11 |
| Pruritus | 52.8% (56/106) | 0.9% (1/106) | 53.8% (57/106) | 11 |
| Rash | 40.5% (420/1038) | 18.9% (208/1100) | 57.9% (586/1012) | 5, 1, 3, 11, 8, 6, 10, 4, 12, 7, 14 |
| **Renal** | | | | |
| Proteinuria | 1.4% (4/287) | 0% (0/287) | 1.4% (4/287) | 14 |
| **Vascular** | | | | |
| Phlebitis | 14.8% (4/27) | 0% (0/27) | 14.8% (4/27) | 2 |
| Thrombosis | 7.4% (26/349) | 11.7% (41/349) | 19.2% (67/349) | 8, 14 |
| **Others** | | | | |
| Asthenia/ Fatigue | 30.2% (232/768) | 3.5% (27/768) | 33.7% (259/768) | 1, 2, 6, 11, 14 |
| Blood albumin decreased | 33.0% (35/106) | 0% (0/106) | 33.0% (35/106) | 11 |
| Fever | 27.9% (160/573) | 1.0% (6/573) | 29.0% (166/573) | 1, 2, 11, 14 |
| Hand-foot syndrome | 22.4% (13/58) | 0% (0/58) | 22.4% (13/58) | 4 |
| Hemorrhage | 17.8% (51/287) | 5.6% (16/287) | 23.3% (67/287) | 14 |
| Hypertension | 8.0% (23/287) | 1.0% (3/287) | 9.1% (26/287) | 14 |
| Infection | 27.3% (105/385) | 19.5% (75/385) | 40.4% (139/344) | 8, 10, 4 |
| ILD-like syndrome | - - | - - | 2.5% (7/282) | 10 |
| Weight decreased | 47.2% (50/106) | 2.8% (3/106) | 50.0% (53/106) | 11 |
| Wound healing complications | 0.7% (2/287) | 0.3% (1/287) | 1.0% (3/287) | 14 |

ILD, interstitial lung disease.

**References:**

1. Aranda E, Manzano JL, Rivera F, Galan M, Valladares-Ayerbes M, Pericay C, et al. (2012) Phase II open-label study of erlotinib in combination with gemcitabine in unresectable and/or metastatic adenocarcinoma of the pancreas: relationship between skin rash and survival (Pantar study). Ann Oncol **23**: 1919-1925.
2. Ardavanis A, Kountourakis P, Karagiannis A, Doufexis D, Tzovaras AA, Rigatos G. (2009) Biweekly gemcitabine (GEM) in combination with erlotinib (ERL): an active and convenient regimen for advanced pancreatic cancer. Anticancer Res **29**: 5211-5217.
3. Bengala C, Sternieri R, Malavasi N, Ponti G, Bertolini F, Zironi S, et al. (2009) Phase II trial of erlotinib in combination with increasing dose of gemcitabine given as fixed dose rate infusion in advanced pancreatic cancer (APC). Available: http://www.asco.org/ASCOv2/Meetings/Abstracts?&vmview=abst_detail_view&confID=63&abstractID=10332. Accessed 7 November 2012.
4. Boeck S, Vehling-Kaiser U, Waldschmidt D, Kettner E, Märten A, Winkelmann C, et al. (2010) Erlotinib 150 mg daily plus chemotherapy in advanced pancreatic cancer: an interim safety analysis of a multicenter, randomized, cross-over phase III trial of the 'Arbeitsgemeinschaft Internistische Onkologie'. Anticancer Drugs 21:94-100.
5. Cheng YJ, Bai CM, Zhang ZJ. (2010) Efficacy of gemcitabine combined with erlotinib in patients with advanced pancreatic cancer. Zhongguo Yi Xue Ke Xue Yuan Xue Bao **32**: 421-423. (in Chinese)
6. Feliu J, Borrega P, Leon A, Lopez-Gomez L, Lopez M, Castro J, et al. (2011) Phase II study of a fixed dose-rate infusion of gemcitabine associated with erlotinib in advanced pancreatic cancer. Cancer Chemother Pharmacol **67**: 215-221.
7. Kim GP, Foster NR, Salim M, Flynn PJ, Moore DF, Zon R, et al. (2011) Randomized phase II trial of panitumumab (P), erlotinib (E), and gemcitabine (G) versus erlotinib-gemcitabine in patients with untreated, metastatic pancreatic adenocarcinoma. Available: http://www.asco.org/ascov2/Meetings/Abstracts?&vmview=abst_detail_view&confID=102&abstractID=82619. Accessed 7 November 2012.
8. Munoz Llarena A, Mane J, Lopez-Vivanco G, Ruiz de Lobera A, Sancho A, Iruarrizaga E, et al. (2011) Gemcitabine (G) fixed-dose-rate infusion (FDR) plus erlotinib (E) in patients with advanced pancreatic cancer (APC). Available: http://www.asco.org/ASCOv2/Meetings/Abstracts?&vmview=abst_detail_view&confID=103&abstractID=71207. Accessed 7 November 2012.
9. Milella M, Vaccaro V, Sperduti I, Bria E, Gelibter A, Mansueto G, et al. (2010) Phase II study of erlotinib (E) combined with fixed dose-rate gemcitabine (FDR-Gem) as first-line treatment for advanced adenocarcinoma of the pancreas (PDAC). Available: http://www.asco.org/ASCOv2/Meetings/Abstracts?&vmview=abst_detail_view&confID=74&abstractID=51951. Accessed 7 November 2012.
10. Moore MJ, Goldstein D, Hamm J, Figer A, Hecht JR, Gallinger S, et al. (2007) Erlotinib plus gemcitabine compared with gemcitabine alone in patients with advanced pancreatic cancer: a phase III trial of the National Cancer Institute of Canada Clinical Trials Group. J Clin Oncol **25**: 1960-1966.
11. Okusaka T, Furuse J, Funakoshi A, Ioka T, Yamao K, Ohkawa S, et al. (2010) Phase II study of erlotinib plus gemcitabine in Japanese patients with unresectable pancreatic cancer. Cancer Sci **102**: 425-431.
12. Philip PA, Goldman BH, Ramanathan RK, Lenz HJ, Lowy AM, Whitehead RP, et al. (2012) Phase I randomized phase II trial of gemcitabine, erlotinib, and cixutumumab versus gemcitabine plus erlotinib as first-line treatment in patients with metastatic pancreatic cancer (SWOG-0727). Available: http://www.asco.org/ASCOv2/Meetings/Abstracts?&vmview=abst_detail_view&confID=115&abstractID=87986. Accessed 7 November 2012.
13. Stuebs P, Habermann P, Zierau K, Schuette K, Fahlke J, Ridwelski K, et al. (2010) First-line therapy for advanced pancreatic cancer with gemcitabine and docetaxel versus gemcitabine and erlotinib: A multivariate matched pair analysis. Available: http://www.asco.org/ASCOv2/Meetings/Abstracts?&vmview=abst_detail_view&confID=74&abstractID=52331. Accessed 7 November 2012.
14. Van Cutsem E, Vervenne WL, Bennouna J, Humblet Y, Gill S, Van Laethem JL, et al. (2009) Phase III trial of bevacizumab in combination with gemcitabine and erlotinib in patients with metastatic pancreatic cancer. J Clin Oncol **27**: 2231-2237.
